# Outcomes of Kidney Transplant Recipients Versus Non-Recipients in the Intensive Care Unit: A Systematic Review and Meta-Analysis

**DOI:** 10.3390/jcm14072284

**Published:** 2025-03-27

**Authors:** Lattawat Eauchai, Wannasit Wathanavasin, Pajaree Krisanapan, Supawit Tangpanithandee, Supawadee Suppadungsuk, Charat Thongprayoon, Wisit Cheungpasitporn

**Affiliations:** 1 Department of Anatomy, Faculty of Medicine, Siriraj Hospital, Mahidol University, Bangkok 10700, Thailand; eauchai.lattawat@gmail.com; 2 Division of Nephrology and Hypertension, Department of Medicine, Mayo Clinic, Rochester, MN 55905, USA; wannasit.medical@mfu.ac.th (W.W.); cheungpasitporn.wisit@mayo.edu (W.C.); 3 Nephrology Unit, Department of Medicine, Charoenkrung Pracharak Hospital, Bangkok Metropolitan Administration, Bangkok 10120, Thailand; 4 Division of Nephrology, Department of Internal Medicine, Faculty of Medicine, Thammasat University, Pathum Thani 12120, Thailand; 5 Division of Nephrology, Department of Internal Medicine, Thammasat University Hospital, Pathum Thani 12120, Thailand; 6 Chakri Naruebodindra Medical Institute, Faculty of Medicine Ramathibodi Hospital, Mahidol University, Samut Prakan 10540, Thailand; tangpanithandee.s@gmail.com (S.T.); supawadee.sup@mahidol.ac.th (S.S.)

**Keywords:** kidney transplantation, critical care, ICU outcomes, mortality, renal replacement therapy, mechanical ventilation, SOFA score, systematic review, meta-analysis

## Abstract

**Background/Objectives:** With the growing population of kidney transplant recipients (KTRs) in intensive care units (ICUs), understanding their prognostic outcomes is critical. As conflicting findings exist, we aim to systematically evaluate and meta-analyze ICU outcomes in kidney transplant recipients compared to non-recipients. **Methods:** We conducted a comprehensive search of the PubMed, Embase, and Cochrane databases, from inception through 23 December 2024, to identify relevant studies comparing the outcomes of KTRs and non-transplant ICU patients. Odds ratios (ORs) with 95% confidence intervals (CIs) were calculated for dichotomous outcomes, and weighted mean differences (WMDs) were calculated for continuous outcomes. The risk of bias was assessed using the ROBINS-I V2 tool. The study protocol was registered in the International Prospective Register of Systematic Reviews (CRD42024595104). **Results:** Seven studies, including 12,062 patients, were analyzed. Demographics, including age and sex, were comparable across groups. No statistically significant associations were found for overall mortality (OR: 1.82, 95% CI: 0.79 to 4.16), ICU mortality (OR: 1.06, 95% CI: 0.45 to 2.48), or 28/30-day mortality (OR: 2.06, 95% CI: 0.30 to 14.10) in KTRs, though there was a trend suggesting a potential increase in the odds of overall mortality. KTRs tended to have longer ICU stays (WMD: +1.96 days, 95% CI: 0.81–3.11) and higher Sequential Organ Failure Assessment (SOFA) scores (WMD: +0.79, 95% CI: −0.78–2.36), but these findings did not reach statistical significance. One study reported higher 1-year and 5-year mortality for KTRs. Sensitivity analyses revealed one influential study. Begg’s test for overall mortality suggested non-significant publication bias (*p* = 1.0). **Conclusions:** KTRs in ICUs are at significantly higher risk for long-term mortality, emphasizing the need for tailored critical care strategies and long-term management. Future research should focus on standardizing methodologies, reducing heterogeneity, and addressing gaps in data to improve evidence-based care for this vulnerable population.

## 1. Introduction

The kidney is the most commonly transplanted solid organ worldwide [1], and kidney transplantation (KT) remains the optimal treatment for end-stage kidney disease (ESKD) [2]. Compared to patients maintained on dialysis, kidney transplant recipients (KTRs) consistently show improved life expectancy and quality of life [2,3]. However, despite these advantages, studies over the past three decades have reported a higher rate of intensive care unit (ICU) admission, a higher risk of mortality, and worse outcomes in KTRs compared to the general population [4,5,6]. This increased risk has been attributed to several factors, including immunosuppression from required medications, comorbidities, and multi-system deterioration resulting from chronic kidney disease prior to transplantation.

Some studies have proposed varying and, at times, conflicting explanations for these worse outcomes in KTRs. While some suggest that the immunosuppressive medications used in this population are a major contributing factor [7,8], others emphasize that the management of acute conditions and comorbidities may play a more significant role than immunosuppression or the transplantation status itself [9]. Interestingly, a recent study found that the need for ICU care, regardless of transplantation status, is more strongly correlated with higher mortality [6].

In addition, variability in study populations and ICU settings has contributed to the discrepancies in reported outcomes over the decades. An earlier study broadly examined the outcomes of KTRs admitted to the ICU for various indications, including infections, respiratory failure, blood loss, and postoperative monitoring [4]. More recently, studies have narrowed their focus to specific subpopulations of KTRs, such as those with COVID-19 infections or those undergoing cardiac surgery. For example, there are studies comparing postoperative outcomes among KTRs admitted to the ICU immediately after transplant surgery (direct ICU admission) versus those who were admitted to a general floor after surgery but were later transferred to the ICU (interval admission) [10,11]. These differences in study populations and admission timings highlight the limited data available on how to effectively manage KTRs in critical care settings.

With the increasing prevalence of ESKD and expanded listing criteria for transplantation, the number of kidney transplant candidates has grown steadily over recent decades, leading to a proportional increase in the population of KTRs [12,13]. Consequently, the number of KTRs requiring ICU care has also risen [5,11].

This growing population of KTRs and the discrepancies in reported outcomes and associated factors necessitate further investigation to better understand the prognostic outcomes of KTRs in critical care settings. Therefore, we aim to perform a systematic review and meta-analysis to compare the outcomes of KTRs with those of non-transplant patients requiring ICU care, and to identify potential predictive factors for these outcomes.

## 2. Materials and Methods

### 2.1. Search Strategy

This systematic review protocol was registered with the International Prospective Register of Systematic Reviews (PROSPERO; CRD42024595104). The Preferred Reporting Items for Systematic Reviews and Meta-Analyses (PRISMA) protocol and the Cochrane Collaboration Handbook were followed for the reporting of this review [14,15]. The PRISMA 2020 checklist is provided in Supplementary Table S1. Two investigators independently conducted a systematic search using the Ovid search system in the following databases, from inception to 23 December 2024: the MEDLINE, EMBASE, and Cochrane databases. The search terms included “Kidney transplant OR Renal transplant” AND “Death OR Mortality OR Acute kidney injury OR RRT dependency” AND “Intensive care OR ICU OR Critically ill”. The detailed search strategy is provided in Supplementary Table S2. Our search also included any relevant conference abstracts and clinical trial registries. We performed this search to include only human studies with no language restriction. In addition, we conducted a manual search of the references in the included studies to identify any additional records.

### 2.2. Eligibility Criteria

Studies were included if they were observational studies or clinical trials reporting the outcomes of adult patients aged ≥ 18 years old who had undergone KT and were admitted to the ICU during the study period. The studies had to report at least one of the following outcomes: mortality, ICU length of stay, need for mechanical ventilation (MV), need for renal replacement therapy (RRT), duration of RRT during ICU stay, need for inotropic drugs, acute kidney injury (AKI), and resultant RRT dependency. The included studies had to compare the outcomes of KT recipients with non-transplant patients admitted to the ICU as a control group. Studies were excluded if they indicated that their KT recipients underwent simultaneous multi-organ transplantation instead of isolated kidney transplantation, indicated that their control subjects had pre-existing chronic renal failure, did not report outcomes of interest, were case reports, were case series, or were other types of publications (a review article, letter to the editor, or technical note).

### 2.3. Study Selection

According to the study eligibility criteria, two reviewers (L.E. and P.K.) independently screened abstracts and titles after removing duplicate publications. Full-text inspection of the studies was performed to determine final eligibility. In the case of the two reviewers reaching a different decision about a study’s eligibility, the final decision was obtained by a discussion involving the participation of all of the authors.

### 2.4. Data Extraction and Quality Assessment

The following variables were extracted from each included study and input into the standardized data collection form by each investigator (L.E. and P.K.): study title, names of authors, year of publication, type of study, country of study, study duration, admission period, ICU type, population, comparator, number of patients, sex, age, body mass index (BMI), level of creatinine (Cr) at baseline and admission, eGFR, 24 h urine protein, albumin level, cause of ESKD, donor-related characteristics (e.g., type of donor, age, and HLA mismatches), comorbidities (e.g., diabetes mellitus, hypertension, history of smoking, etc.), reason for ICU admission, time from transplantation to ICU admission, severity scores at admission (including the Simplified Acute Physiology Score (SAPS), the Sequential Organ Failure Assessment (SOFA) score, and the Acute Physiology And Chronic Health Evaluation (APACHE) score), and outcomes including mortality, ICU length of stay, the need for RRT and duration, the need for inotropic support, the need for mechanical ventilation, and new-onset infection in the ICU. We aimed to extract the odds ratio (OR) for categorical outcomes of interest and mean differences for continuous outcomes by comparing KTRs to non-recipient patients in each study. If studies did not directly report the OR, we calculated it using the number of patients with and without the outcomes among KTRs and non-recipients. The methodological quality of each study was assessed by two independent investigators (L.E. and P.K.). The Grading of Recommendations Assessment, Development, and Evaluation (GRADE) methodology was used to assess the quality of evidence from the included studies [15,16]. The Risk of Bias In Non-randomized Studies—of Interventions (ROBIN-I) V2 tool was used to assess the risk of bias in cohort and non-randomized controlled studies [17]. Disagreement between these two investigators was settled by the third investigator (W.W.)

### 2.5. Statistical Analysis

All statistical analyses were performed using the Stata statistical package version 18 (StataCorp, College Station, TX, USA). A two-tailed *p* value of less than 0.05 was considered statistically significant. We conducted a meta-analysis to calculate the pooled estimates in each outcome of interest, following the methods suggested by DerSimonian and Laird, using the random-effects model [18]. When the incidence of an outcome was zero in a study, a continuity correction of 0.5 was added to the number of incident cases before statistical analysis. Pooled odds ratios with corresponding 95% confidence intervals (CIs) were calculated for categorical outcomes, and weighted mean differences (WMDs) were calculated for continuous outcomes.

The presence of heterogeneity among the effect sizes of individual studies was assessed through Cochrane’s Q test and the I^2^ index. I^2^ values of 25%, 50%, and 75% or higher represent a low, moderate, and high degree of heterogeneity, respectively [15]. Due to insufficient data, we could not perform pre-specified subgroup analysis for primary outcomes based on patient baseline characteristics, severity scores, and the type of ICU admission. We also performed a sensitivity analysis using the leave-one-out method, removing one study at a time to assess the robustness of the meta-analysis results. The results of all meta-analyses are visualized as forest plots, and funnel plots were generated to detect publication bias. Egger’s test and Begg’s test were used to assess for publication bias [19,20], and a *p* value of less than 0.05 indicates significant publication bias.

## 3. Results

### 3.1. Characteristics of Included Studies

**Figure 1:**
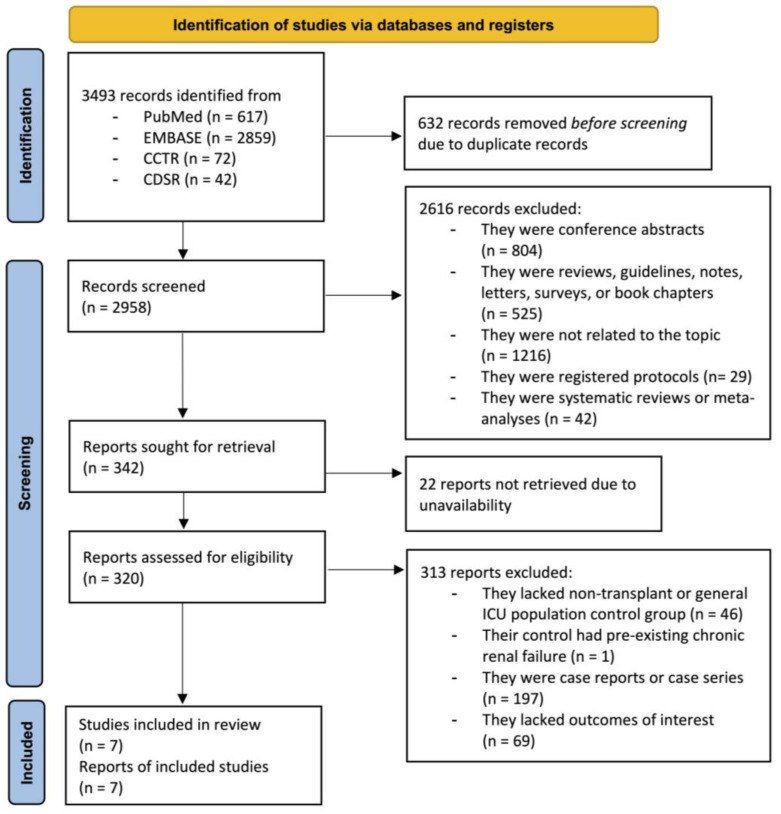
PRISMA flow of search methodology and selection process; CCTR, Cochrane Central, Register of Controlled Trial; CDSR, Cochrane Database of Systematic Review.

A total of 3493 potentially relevant articles were identified through our comprehensive search in the PubMed, EMBASE, and Cochrane Library databases, of which 632 were duplicates. The remaining 2958 articles were screened for titles and abstracts, resulting in the exclusion of 2616 articles based on publication type, missing outcomes or populations of interest, or lack of topic relevance. Additionally, 22 articles were not accessible due to the absence of full-text availability. A total of 320 articles were then subjected to full-text review, with 313 being excluded. The reasons for exclusion are detailed in Figure 1. Finally, seven studies [21,22,23,24,25,26,27], investigating a total of 12,062 patients, were thoroughly reviewed and included for the systematic review and meta-analysis (Figure 1).

The included articles were published from 1995 to 2021. The detailed characteristics and specific data of each article are listed in Table 1. Among the seven selected studies, five were retrospective cohort studies, two were ambispective cohort studies, and the remaining study was a prospective cohort study. Six studies exclusively aimed to study the outcomes of KT recipients (KTRs) in the ICU [21,22,23,24,26,27], while the one remaining study focused on the postoperative outcomes of recipients who underwent cardiac surgery, but nonetheless reported ICU-related outcomes [25]. Three studies indicated their ICU types, including one study in a surgical ICU [21], one study in a medical ICU [22], and one study in a nephrology ICU [26], while the four remaining studies did not specify the ICU setting [23,24,25,27]. Five studies compared KT recipients with non-transplant patients [22,24,25,26,27], while the two other studies indicated the general ICU population as a control group [21,23]. One study specifically examined patients with COVID-19 infection [24]. Overall, the mean age of KTRs was 49.37 ± 14.4 years, while the mean age of the control population was 54.32 ± 19.83 years. In terms of sex, 65.32% of KTRs and 60.7% of the control population were male.

Two studies were conducted with two groups of KTRs [21,26]: one group admitted to the ICU immediately after or in the same visit as for the kidney transplant surgery (defined as the postoperative KT surgery group), and another group admitted in the later admission different to that for the transplant surgery (defined as the later admission group). Three studies consisted of patients with later ICU admission only [22,24,25]. Two studies did not indicate the timeframe of ICU admission based on KT surgery, and could not classify the participants into these subgroups [23,27].

### 3.2. Methodological Quality

The GRADE approach was followed for assessing the certainty of evidence (Supplementary Table S3). In addition, the ROBIN-I V2 tool was used to identify and assess the risk of bias in included studies. Of all the included studies, 71.43% were rated as having a moderate risk of bias, and the remaining 28.57% were rated as having a low risk of bias. The risks of bias due to confounding and missing data were the main contributors to the rating of the study determined to have a moderate risk of bias. A description of the ROBIN-I V2 tool evaluation details for each study are provided in Supplementary Figure S1.

### 3.3. ICU Outcomes in KTRs Versus Non-Recipients (Table 2)

#### 3.3.1. Mortality Outcomes

##### Overall Mortality

**Figure 2:**
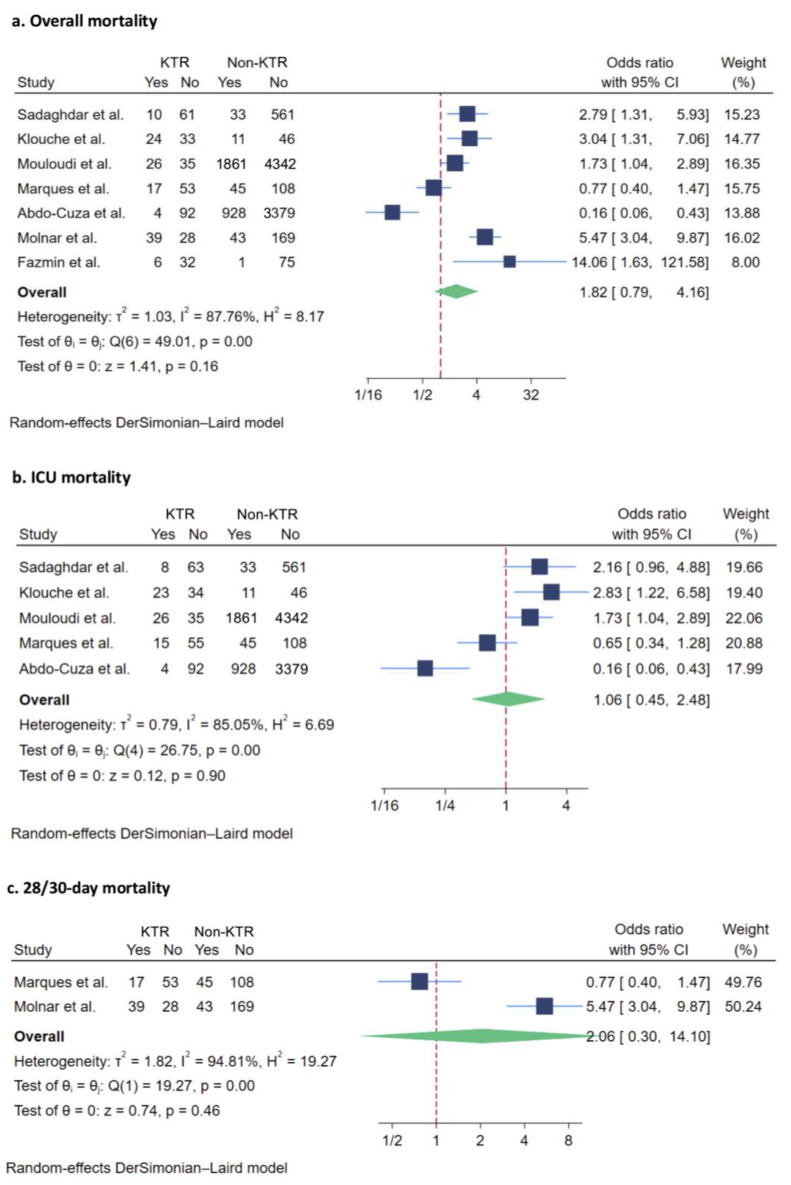
Forest plot visualizing pooled odds ratio of mortality outcomes of kidney transplant recipients compared to non-recipients [21,22,23,24,25,26,27], including: (**a**) overall mortality; (**b**) ICU mortality; and (**c**) 28/30-day mortality. Studies are identified by name of first author. KTR, kidney transplant recipient; CI, confidence interval; ICU, intensive care unit; dark blue square: represents the point estimate of the effect from individual studies. The size of the square often reflects the weight of the study in the meta-analysis, with a larger square indicating greater weight; light blue line: represents 95% CI; red dashed line: represents null effect; green rhombus: represents the summarized effect estimate from the combined studies. The width of the diamond provides an idea about the precision of the estimate. A wider diamond suggests less precision, while a narrower diamond indicate more precision.

Mortality outcomes were the most reported outcomes across all studies. All seven studies reported overall mortality, which included all reported mortality during hospitalization and short-term follow-up. Of the 12,062 patients evaluated, the pooled analysis demonstrated a non-significant trend toward an increase in overall mortality among KTRs compared to non-recipients (OR: 1.82, 95% CI: 0.79 to 4.16; I^2^ = 87.76%) (Figure 2a).

**Table 2:** Summary of results for outcomes of interest.

Outcomes	No. of Studies	No. of Patients			Odds Ratio (95% CI)	I^2^ (%)
		Total	KTRs	Non-KTRs		
Mortality outcomes						
Overall mortality	7	12,062	460	11,602	1.82 (0.79–4.16)	87.76
ICU mortality	5	11,669	355	11,314	1.06 (0.45–2.48)	85.05
28/30-day mortality	2	502	137	365	2.06 (0.30–14.10)	94.81
1-year mortality	1	114	38	76	6.49	N/A
5-year mortality	1	114	38	76	5.75	N/A
ICU-specific outcomes						
ICU LOS	6	7659	364	7295	+1.96 days * (0.81–3.11)	0
Need for RRT	3	616	175	441	2.20 (0.53–9.07)	91.38
Need for inotropic drugs	3	616	175	441	0.78 (0.52–1.16)	0
Need for MV	2	502	137	365	0.73 (0.33–1.62)	66.35
New infection	1	279	67	212	0.64 (0.34–1.20)	N/A
ICU severity scoring system						
SOFA	2	337	127	210	+0.79 * (−0.78–2.36)	0

CI, confidence interval; N/A, not available; KTRs, kidney transplant recipients; ICU, intensive care unit; LOS, length of stay; RRT, renal replacement therapy; MV, mechanical ventilation; SOFA, Sequential Organ Failure Assessment score; *, reported as weighted mean difference (WMD).

##### ICU Mortality

Following the overall mortality, the ICU mortality was the second most commonly reported outcome from five studies with 11,669 patients [21,23,26,27]. The ICU mortality risk was non-significant in KTRs compared to the non-transplant group (OR: 1.06, 95% CI: 0.45 to 2.48; I^2^ = 85.05%) (Figure 2b).

##### Twenty-Eight/Thirty-Day Mortality

Two studies, comprising 502 patients, reported 28/30-day mortality [24,26]. There was no statistically significant association between increased mortality and KTRs (OR: 2.06, 95% CI: 0.30 to 14.10; I^2^ = 94.81%) (Figure 2c).

##### One-Year Mortality

For long-term mortality, only one study, with 114 patients, reported 1-year mortality, with a higher incidence in the KTR group (OR: 6.49, 95% CI: 1.61 to 26.14) [25].

##### Five-Year Mortality

For mortality at 5 years, only one study, comprising of 114 patients, reported a higher mortality rate in KTRs compared to non-recipients (OR: 5.75, 95% CI: 2.07 to 15.93) [25].

#### 3.3.2. ICU-Specific Outcomes

##### ICU Length of Stay

**Figure 3:**
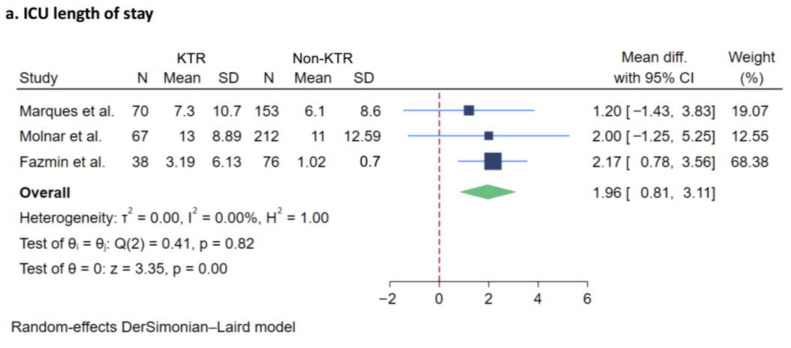

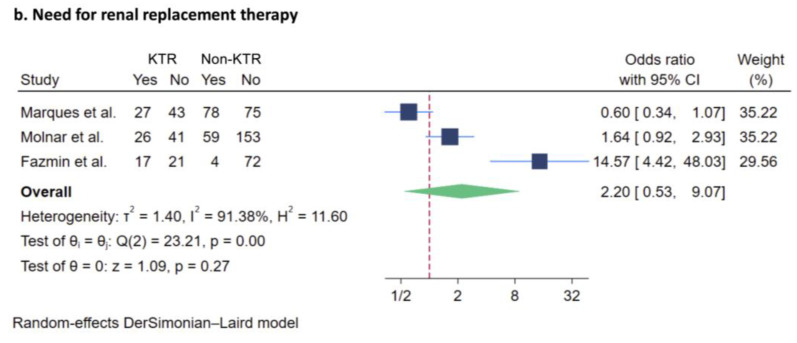
Forest plot visualizing pooled odds ratio of ICU-specific outcomes of kidney transplant recipients compared to non-recipients [24,25,26], including (**a**) ICU length of stay, and (**b**) need for renal replacement therapy. Studies are identified by name of first author. KTR, kidney transplant recipient; CI, confidence interval; ICU, intensive care unit; dark blue square: represents the point estimate of the effect from individual studies. The size of the square often reflects the weight of the study in the meta-analysis, with a larger square indicating greater weight; light blue line: represents 95% CI; red dashed line: represents null effect; green rhombus: represents the summarized effect estimate from the combined studies. The width of the diamond provides an idea about the precision of the estimate. A wider diamond suggests less precision, while a narrower diamond indicate more precision.

The ICU length of stay (LOS) in KTRs was reported in six studies comprising 7659 patients, with average value ranging from 3.19 to 13.3 days [21,22,23,24,25,26,27]. Among the studies including a postoperative KT group, the average LOS ranged from 3.6 to 5.1 days [21,26]. In comparison, the later admission group was reported to have an average LOS range of 3.19 to 13 days [21,22,24,25,26]. Three studies reported the ICU LOS for both KTRs and the control group, with the average LOS in the control group ranging from 1.02 to 11 days [24,25,26]. An analysis was conducted with the data from these three studies. Our meta-analysis revealed a trend toward an increase in the ICU LOS in KTRs compared to non-recipients, though this was non-significant (WMD + 1.96 days, 95% CI 0.81 to 3.11). The I^2^ value was 0% (Figure 3a).

##### Need for Renal Replacement Therapy

Three studies reported the need for RRT, with a total of 616 participants evaluated [24,25,26]. There was no significant difference in the need for RRT of KTRs compared to non-recipients (OR: 2.20, 95% CI 0.53 to 9.07). The I^2^ was 91.38% (Figure 3b).

##### Need for Inotropic Support

**Figure 4:**
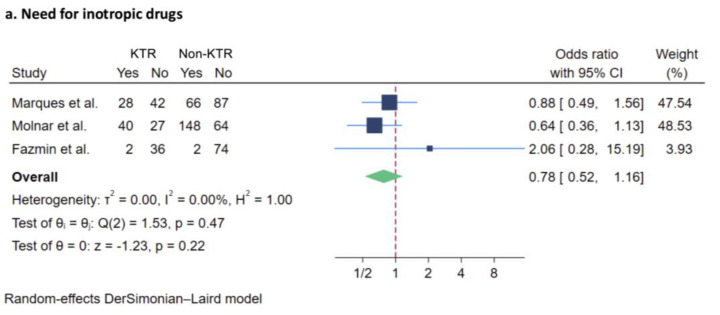

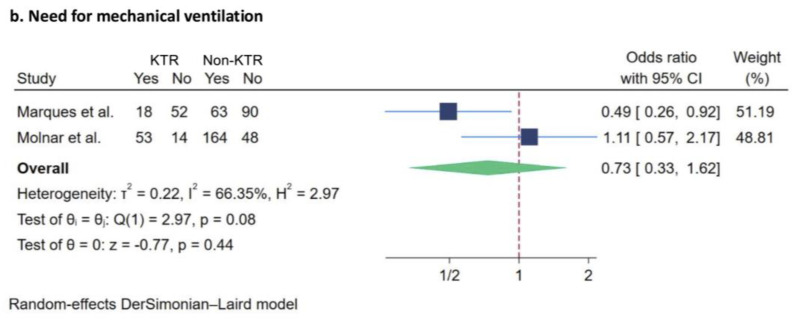
Forest plot visualizing pooled odds ratio of ICU-specific outcomes of KTRs compared to non-recipients [24,25,26], including (**a**) need for inotropic drugs, and (**b**) need for mechanical ventilation. Studies are identified by name of first author. KTR, kidney transplant recipient; CI, confidence interval; ICU, intensive care unit; dark blue square: represents the point estimate of the effect from individual studies. The size of the square often reflects the weight of the study in the meta-analysis, with a larger square indicating greater weight; light blue line: represents 95% CI; red dashed line: represents null effect; green rhombus: represents the summarized effect estimate from the combined studies. The width of the diamond provides an idea about the precision of the estimate. A wider diamond suggests less precision, while a narrower diamond indicate more precision.

Of 616 patients evaluated in three studies [24,25,26], there was no significant difference in the need for inotropic support between KTRs and non-recipients (OR: 0.78, 95% CI 0.52 to 1.16) The I^2^ value was 0% (Figure 4a).

##### Need for Mechanical Ventilation

Two studies, comprising 502 participants, reported the need for mechanical ventilation for KTRs and non-recipients [24,26]. There was no significant difference in the need for mechanical ventilation between KTRs and non-recipients (OR: 0.73, 95% CI: 0.33 to 1.62). The I^2^ value was 66.35% (Figure 4b).

##### New-Onset Infection in the ICU

One study, with a total of 279 patients, revealed the difference in the new infection rate in the ICU between groups [24]. The result showed no significant difference in the rate of infection between KTRs and non-recipients (OR: 0.64, 95% CI: 0.34 to 1.20) [24].

#### 3.3.3. ICU Severity Scoring System

**Figure 5:**
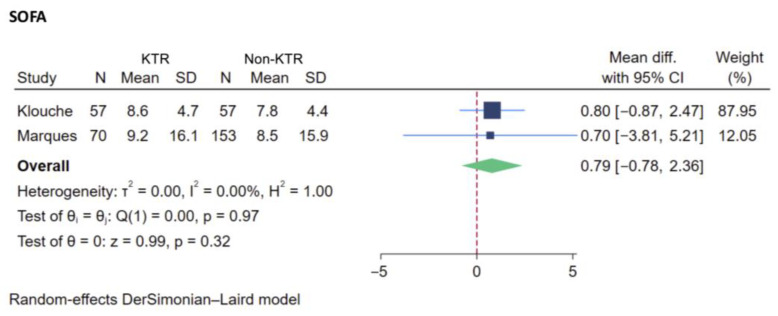
Forest plot visualizing difference in SOFA score of kidney transplant recipients compared to non-recipients [22,26]. Studies are identified by name of first author. SOFA, Sequential Organ Failure Assessment score; KTR, kidney transplant recipient; Mean diff., mean difference; CI, confidence interval; dark blue square: represents the point estimate of the effect from individual studies. The size of the square often reflects the weight of the study in the meta-analysis, with a larger square indicating greater weight; light blue line: represents 95% CI; red dashed line: represents null effect; green rhombus: represents the summarized effect estimate from the combined studies. The width of the diamond provides an idea about the precision of the estimate. A wider diamond suggests less precision, while a narrower diamond indicate more precision.

There was variability among the studies in terms of their reported severity scores, which included the Simplified Acute Physiology Score (SAPS), the Sequential Organ Failure Assessment (SOFA) score, and the Acute Physiology And Chronic Health Evaluation (APACHE) score. The SOFA score was the only severity score reported in both KTRs and non-recipients, from two studies with a total of 337 patients, permitting an analysis of the difference in this score between the two groups [22,26]. There was no statistically significant increase in the SOFA score between KTRs and non-recipients (WMD +0.79, 95% CI −0.78 to 2.36). The I^2^ value was 0% (Figure 5). There were insufficient data to conduct further subgroup analysis for the SOFA score or an analysis for other severity scores, e.g., the SAPS or APACHE score.

### 3.4. Sensitivity Analysis

In order to ensure the reliability of the present meta-analysis, we performed sensitivity analyses using the leave-one-out method for overall and ICU mortality outcomes. For overall mortality, one study was identified as the most influential on the pooled ORs [27]. Details of these sensitivity analyses are provided in Supplementary Table S4.

### 3.5. Assessment of Publication Bias

**Figure 6:**
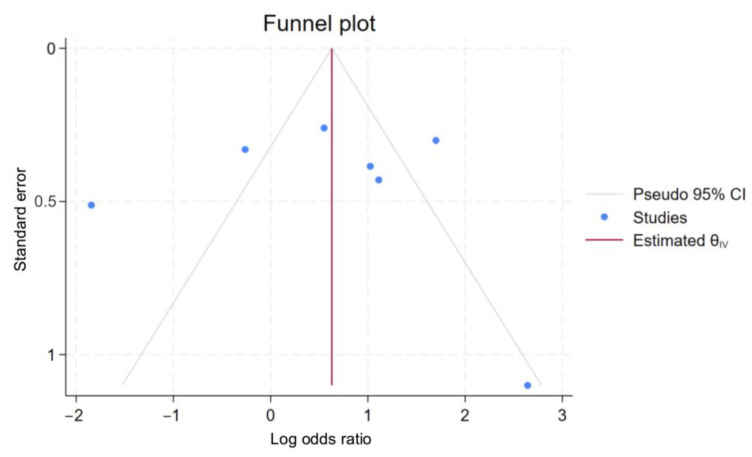
Funnel plot for assessment of publication bias for overall mortality of kidney transplant recipients compared to non-recipients; CI, confidence interval.

Publication bias was evaluated through visual inspection of funnel plots, Egger’s regression test, and Begg’s test. The funnel plot for overall mortality in the studies included in the meta-analysis was asymmetrical (Figure 6). However, Egger’s test did not indicate significant publication bias (*p* = 0.4345). In addition, evaluation by Begg’s test revealed non-significant publication bias (*p* = 1.00).

## 4. Discussion

In our systematic review and meta-analysis, we identified high heterogeneity in the ICU outcomes of KTRs compared to non-transplant patients or the general population reported in studies published over three decades. For short-term outcomes, including mortality, ICU length of stay, the need for ICU intervention, such as mechanical ventilation, renal replacement therapy, or inotropic support, and the SOFA score, there was no statistically significant increase in the odds of these outcomes in KTRs with high heterogeneity. For long-term outcomes, 1-year and 5-year mortality were reported to be higher in KTRs. Though there was no statistically significant association between kidney transplantation status and the majority of these outcomes, the high heterogeneity observed could suggest the probability that varying factors, including patient demographics, study designs, or changes and development in patient care over the decades, might be responsible for such observations.

Over the past two decades, the ICU admission rate among KTRs has continuously increased [7,21,22], reflecting the growing size of this population and advancements in transplantation and critical care. However, some studies report lower admission rates [23], likely due to differences in admission criteria and institutional practices for KTRs. Although most studies were conducted in North America and Europe, recent data from Korea have also shown a rising incidence of ICU admissions among KTRs and other organ transplant recipients [5]. These findings suggest a global trend of increasing ICU care needs for KTRs.

The growing KTR population and conflicting findings regarding outcomes necessitate a better understanding of the characteristics and prognoses of KTRs in the ICU. Our study is the first meta-analysis to compare the ICU outcomes of KTRs with those of non-transplant patients, providing valuable insights into this critical care population.

Mortality is the most reported outcome for KTRs versus non-recipients in the ICU. Several studies have reported higher mortality rates among KTRs compared to the general ICU population [7,21,22,23]. The suggested reasons for such an observation include prolonged deterioration in renal function and the use of immunosuppressive drugs, which increase susceptibility to severe infections [8,9]. These factors likely contribute to the elevated risk of multi-organ dysfunction and mortality in this population. However, conflicting evidence exists. Some studies have shown comparable mortality rates between KTRs and non-recipients or the general ICU population [23], while others have found no association between immunosuppressive drug use and mortality in transplant recipients, including KTRs [11]. These inconsistencies underscore the complexities of managing critically ill KTRs. Our meta-analysis revealed a non-significant increase in the odds of short-term mortality, including overall, ICU, and 28/30-day mortality, in KTRs. The reported results showed high heterogeneity in all these outcomes, reflecting inconsistencies across studies (Table 2). For overall mortality, sensitivity analysis identified one influential study (Abdo-Cuza et al.), which significantly affected the pooled estimates [27]. Removing this study revealed a significant increase in overall mortality for KTRs [27]. Abdo-Cuza et al. reported comparable mortality rates between KTRs and matched non-recipients, but provided limited demographic and ICU-related data. The study’s focus on hospital-associated infections as the primary outcome and its large sample size likely influenced the pooled analysis [27]. It is worth noting that each of the included studies was conducted on KTRs with different time periods post kidney transplantation, which could have caused differential characterization of the risk factors for critical illness. For ICU mortality, the high heterogeneity was likely due to differences in ICU management protocols and patient populations, e.g., postoperative patients or patients with a medical condition unrelated to KT. Meanwhile, the substantial heterogeneity in 28/30-day mortality could be due to later complications of KT. Postoperative complications such as infections, cardiovascular events, and graft rejection disproportionately affect KTRs during this period. For long-term outcomes, a previous study revealed a significant increase in 1-year and 5-year mortality among KTRs admitted to the ICU [25], although the data on long-term mortality were insufficient for inclusion of this study in this meta-analysis. This significant finding highlights the importance of long-term monitoring and targeted interventions to improve survival in KTRs.

Previous studies have suggested that the need for certain methods of organ support management, e.g., mechanical ventilation (MV), inotropic support, renal replacement therapy (RRT), etc., is a predictive factor for worse outcomes for KTRs in the ICU [8,22]. However, findings from these studies were inconclusive for some specific management methods. We evaluated these ICU management methods to gain insights for the management of critically ill KTRs. For mechanical ventilation, no significant difference was observed in the need for mechanical ventilation between KTRs and non-recipients. This outcome suggests that transplant status may not significantly influence the risk of respiratory failure, although variability in the study settings and criteria for mechanical ventilation may partially explain the findings. The need for RRT was also found to be higher in KTRs, although this was non-significant. This finding may reflect the higher baseline risk of renal dysfunction in KTRs, particularly under critical care stressors such as sepsis or hemodynamic instability. It is worth noting that the high heterogeneity of this outcome could imply variability between studies. Additionally, the need for inotropic support was similar in both groups, which could suggest differences in cardiovascular profiles or ICU management strategies between the groups.

Interestingly, recent studies comparing survivors and non-survivors among kidney transplant recipients have revealed that the need for critical care management, including mechanical ventilation (MV) and inotropic drugs, was a stronger predictor of worse outcomes than transplantation status itself [6,9]. While these studies compared survivor and non-survivor KTR populations, which differs from our analysis, their findings revealed a worse prognosis in certain patient groups, and suggest a promising approach for future studies. Analyzing KTR populations with varying severity, such as survivors versus non-survivors, could provide valuable insights into ICU prognosis for this patient group.

ICU severity scores are useful tools for predicting patient outcomes, and warrant close monitoring and critical management for patients who are at risk. Within the KTR population, the established studies provided conflicting evidence on the application of these severity scores. Two earlier studies revealed non-significant higher SOFA scores for KTRs compared to non-recipients [22,26]. In one of these studies, further subgroup classification of KTR patients into postoperative and later admission subgroups revealed that significantly higher SOFA scores correlated with a higher mortality rate in the later admission group compared to postoperative group and non-recipients [26], suggesting that differences in transplant status might have accounted for observed discrepancy. Comparing the data between survivors and non-survivors within KTR populations, a strong correlation of severity scores, including the APACHE, SAPS, and SOFA scores, with mortality in the non-survivor group was observed in multiple studies [21,22,26]. While Sadaghdar et al. reported a comparable APACHE II score between subgroups of patients with postoperative KT and medical admission, the study also revealed a drastically different correlation with mortality between these two groups. APACHE II was predictive for mortality in the later medical admission group, while there was a lower mortality rate than predicted by APACHE II in the postoperative group. Conversely, Abrol et al. reported higher SOFA and APACHE III scores, but a lower actual mortality rate, in KTRs requiring early admission, compared to KTR patients admitted later [11]. These conflicting findings underscore the necessity to confirm such a correlation and to carefully interpret findings based on study differences. Our analysis revealed slightly higher SOFA scores (+0.79) for KTR patients admitted to the ICU, suggesting a trend toward a greater severity of illness, but the lack of statistical significance limits strong conclusions (Figure 4). The inability to perform a meta-analysis for the APACHE score due to limited data highlights a critical gap in the literature. Collecting standardized severity scores across future studies is essential for further analyses.

We initially aimed to further evaluate these outcomes based on the stratification of patients’ demographics, immunosuppressive regimen and status, and different ICU admission times, between postoperative KT surgery and later admission not relevant to KT surgery. However, there were limited data, due to high variation in data variables across all of the included studies, which prevented further subgroup analysis. Further studies with standardized methodologies and data collection are necessary to better elucidate the characteristics and prognostic outcomes of KTRs in the ICU.

There are insufficient data to conduct an analysis for the reasons for ICU admission, with the absence of data from a control population constituting a major obstacle. However, the majority of the included studies reported sepsis and acute respiratory failure as the most common reasons for ICU admission and mortality for KTRs admitted to the ICU [21,22,23,24,26]. We looked into groups of studies with different ICU admission periods to identify any potential trends or characteristics. For later ICU admission, sepsis and respiratory failure remained the most common reasons for ICU care. In studies comprising both post-KT and later admission groups, it was reported that sepsis and infection were better attributed to the later admission group [21,26]. One study suggested that this finding was likely due to a longer duration of immunosuppressive therapy [21]. However, there are limited data, as subsequent studies did not further examine this correlation. In studies comprising postoperative patients, the main reasons for ICU admission were surgery-related complications, cardiovascular abnormalities, and monitoring [11,21,26]. Abrol et al. extensively studied a postoperative population by comparing those requiring ICU admission immediately after transplant surgery with an interval admission group, which was defined as a patient group requiring ICU transfer later on, but in the same visit as for the transplant surgery. This study reported that the early admission group was more likely to have a higher BMI and, have undergone a concomitant operation, have higher severity scores, including SOFA and APACHE III scores. In addition, a higher percentage of patients in this group required MV. However, there was lower mortality and a shorter MV duration reported in this group compared to the interval admission group. The study suggested that this was due to the patients in the early admission group being more likely to have a shorter recovery duration [11].

Though our analyses revealed several non-significant results, these findings suggest trends in several clinical components, which could be applied in practice to improve patient care, drive clinical knowledge enhancement, and encourage further research. Firstly, the significant increase in 1-year and 5-year mortality underscores the importance of enhanced post-transplant care, focusing on managing comorbidities, optimizing immunosuppression, and preventing chronic complications. Secondly, the potential trends of increased RRT needs, longer ICU stays, and severity score in KTR patients should prompt heightened awareness and resource planning in ICUs managing these patients. Thirdly, there are differences in characteristics and prognosis between KTRs admitted immediately after transplantation and those requiring later ICU admission. For the immediate postoperative period, the short-term mortality rate was lower, but the initial higher severity scores in certain groups highlight the need for vigilant perioperative care, particularly for managing complications during this critical period. For KTRs requiring later ICU admission, higher mortality rates and higher needs for organ support management warrant a personalized approach in managing KTR patients during the maintenance period. Lastly, the variability across studies, as reflected by high heterogeneity, calls for standardized methodologies and reporting to improve the reliability of future meta-analyses.

There are several limitations to our study. The elevated I^2^ values observed for most outcomes suggest variability in the study populations and methodologies, which may affect the consistency of the findings. The included studies reported diverse patient demographics and outcomes, with inconsistencies in severity scoring systems, such as the use of the SOFA, SAPS III, or APACHE II scores. Additionally, the exclusion of several studies, due to a lack of control populations or unclear definitions of study cohorts, resulted in the loss of potentially valuable data that could have strengthened the analysis. The reliance on observational studies further introduced biases and confounding factors, restricting causal inferences and emphasizing the need for prospective, well-designed studies to validate the findings. Moreover, sensitivity analyses revealed that a specific study disproportionately influenced the pooled estimates, likely due to unique population characteristics or ICU protocols. To address this, we emphasize the need for future studies that incorporate standardized data collection methodologies and covariate adjustments, in order to improve the robustness and clinical applicability of findings. Such studies would enhance our understanding of the complex interplay between baseline characteristics, transplant-specific risk factors, and ICU outcomes in KTRs. We believe that this refinement will strengthen context of our findings and offer meaningful guidance for future research directions. Addressing these limitations will enhance the reliability and applicability of future research on KTRs in critical care settings.

## 5. Conclusions

This systematic review and meta-analysis highlights the increased risk of 1-year mortality in KTRs, emphasizing the importance of tailored care strategies during the immediate postoperative period, and of comprehensive long-term follow-up. Our findings underscore the need for interventions aimed at mitigating long-term complications, such as chronic graft dysfunction, infections, and cardiovascular events, which contribute significantly to mortality. Future research should focus on reducing heterogeneity by standardizing methodologies, adopting consistent definitions for outcomes, and incorporating diverse patient populations across multicenter studies. Addressing these gaps will not only improve the reliability and applicability of evidence, but also provide actionable insights to guide clinical decision-making and enhance the overall care of KTRs in critical care settings.

## Figures and Tables

**Table 1:** Characteristics of included studies.

Author (Year)	Sadaghdar et al. [21] (1995)	Klouche et al. [22] (2009)	Mouloudi et al. [23] (2012)	Marques et al. [26] (2015)	Abdo-Cuza et al. [27] (2020)	Molnar et al. [24] (2020)	Fazmin et al. [25] (2021)
Study type	Prospective cohort	Retrospective cohort	Ambispective cohort	Retrospective cohort	Retrospective cohort	Ambispective cohort	Retrospective cohort
Site	USA, single center	France, single center	Greece, single center	Brazil, single center	Cuba, multicenter	USA, multicenter	UK, single center
Study duration, year(s)	1	10	20	1	5	3 months	8
Admission period	Post-transplant and later admission	Later admission	Not specified	Post-transplant and later admission	Not specified	Later admission	Later admission
ICU type	Surgical	Medical	N/A	Nephrology	N/A	N/A	N/A
Population of interest	KTRs	KTRs	KTRs	KTRs	KTRs	KTRs with COVID-19	KTRs who underwent cardiac surgery
Comparator	General ICU population	Non-recipients	General ICU population	Non-recipients	Matched non-recipients	Matched non-recipients with COVID-19	Matched non-recipients who underwent cardiac surgery
Total N	665	114	6264	223	4403	279	114
KTR	71	57	61	70	96	67	38
Control	594	57	6203	153	4307	212	76
Male, n (%)	48 (67.61)	30 (52.63)	50 (81.97)	41 (58.57)	N/A	N/A	25 (65.79)
Age, year	47 ± 15	51.6 ± 14.1	45.5 ± 12.5	52.2 ± 12.8	44.8 ± 14.58	N/A	63, (56.3–67) *
Body mass index, kg/m^2^	N/A	N/A	N/A	N/A	N/A	N/A	30.1 (25.6–34.5)
eGFR at admission, mL/min/1.73 m^2^	N/A	N/A	N/A	17.3 ± 15.4	N/A	N/A	N/A
Baseline Cr, mmol/L	N/A	232 ± 140	N/A	N/A	N/A	N/A	165.19 ± 127.6
Cr at admission, mmol/L	N/A	303 ± 158	N/A	468.52 ± 256.36	N/A	N/A	N/A
Albumin, g/dL	N/A	2.7 ± 0.6	N/A	2.4 ± 1.5	N/A	N/A	N/A
Donor-related characteristics							
Living donor, n (%)	N/A	3 (5.26)	21 (34.3)	17 (24.29)	N/A	N/A	N/A
Donor age, year	N/A	45.8 ± 15.6	N/A	44 ± 16	N/A	N/A	N/A
HLA mismatches	N/A	2.9 ± 0.9	N/A	3 ± 1.5	N/A	N/A	N/A
Comorbidities							
Diabetes mellitus, n (%)	N/A	N/A	N/A	24 (34.29)	N/A	N/A	11 (28.95)
Hypertension, n (%)	N/A	N/A	N/A	N/A	N/A	N/A	29 (76.32)
History of smoking, n (%)	N/A	N/A	N/A	N/A	N/A	N/A	19 (50)
CAD, n (%)	N/A	N/A	N/A	N/A	N/A	N/A	N/A
CHF, n (%)	N/A	N/A	N/A	N/A	N/A	N/A	N/A
Arrhythmia, n (%)	N/A	N/A	N/A	N/A	N/A	N/A	3 (7.9)
COPD	N/A	N/A	N/A	N/A	N/A	N/A	5 (13.16)
Asthma	N/A	N/A	N/A	N/A	N/A	N/A	3 (7.9)
Time from transplantation to ICU admission, months	23 ± 30	32.95 ± 46.52	18.5, (0–216) ^++^	6.37, (0–357.95) ^++^	N/A	N/A	N/A
SOFA	N/A	8.6 ± 4.7	8.5 ± 3.5	9.2 ± 16.1	N/A	N/A	N/A
APACHE II	19.4 ± 6	N/A	20 ± 5.7	N/A	13.76 ± 8.01	N/A	N/A

N/A, data not available; *, median, IQR; ^++^, range; ICU, intensive care unit; KTR, kidney transplant recipient; eGFR, estimated glomerular filtration; Cr, creatinine; CAD, coronary artery disease; CHF, congestive heart failure; COPD, chronic obstructive pulmonary disease; SOFA, Sequential Organ Failure Assessment; APACHE, Acute Physiology and Chronic Health Evaluation.

## Data Availability

The data supporting this study can be found in the original publications, reports, and preprints referenced in the citations.

## Supplementary Materials

The following supporting information can be downloaded at https://www.mdpi.com/article/10.3390/jcm14072284/s1: Table S1: PRISMA checklist; Table S2: Search terms; Table S3: Summary of findings and certainty assessment in accordance with GRADE framework; Table S4: Sensitivity analysis; Figure S1: Summary of Risk of Bias in Non-randomized Studies—of Interventions (ROBIN-I) for included studies.

## Abbreviations

The following abbreviations are used in this manuscript:

AKI	Acute kidney injury
APACHE	Acute Physiology And Chronic Health Evaluation score
BMI	Body mass index
CI	Confidence interval
Cr	Creatinine
eGFR	Estimated glomerular filtration rate
ESKD	End-stage kidney disease
HLA	Human leukocyte antigen
ICU	Intensive care unit
KT	Kidney transplantation
KTR	Kidney transplant recipient
LOS	Length of stay
MV	Mechanical ventilation
RRT	Renal replacement therapy
OR	Odds ratio
ROBINS-I	Risk of Bias in Non-randomized Studies—of Interventions
SAPS	Simplified Acute Physiology Score
SOFA	Sequential Organ Failure Assessment score
WMD	Weighted mean difference
