# Supplementary material for: Outcomes of Kidney Transplant Recipients Versus Non-Recipients in the Intensive Care Unit: A Systematic Review and Meta-Analysis
Source: J Clin Med. 2025 Mar 27;14(7):2284. doi: 10.3390/jcm14072284 (PMC11989273; doi:10.3390/jcm14072284)

Supplementary material

Table S1	PRISMA checklist
Table S2	Search terms
Table S3	Summary of findings and certainty assessment in accordance to GRADE framework.
Table S4	Sensitivity analysis
Figure S1	Summary of Risk of Bias in Non-randomized Studies – of Interventions (ROBIN-I) for included studies

Table S1 PRISMA checklist

Topic	No.	Item	Location where item is reported
**TITLE**			
**Title**	1	Identify the report as a systematic review.	Title (page 1, line 2-3)
**ABSTRACT**			
**Abstract**	2	See the PRISMA 2020 for Abstracts checklist	
**INTRODUCTION**			
**Rationale**	3	Describe the rationale for the review in the context of existing knowledge.	Background (page 2, line 48-83)
**Objectives**	4	Provide an explicit statement of the objective(s) or question(s) the review addresses.	Background (page 2, line 79-83)
**METHODS**			
**Eligibility criteria**	5	Specify the inclusion and exclusion criteria for the review and how studies were grouped for the syntheses.	Eligibility criteria (page 3, line 100-112)
**Information sources**	6	Specify all databases, registers, websites, organisations, reference lists and other sources searched or consulted to identify studies. Specify the date when each source was last searched or consulted.	Search strategy (page 2-3, line 85-99)
**Search strategy**	7	Present the full search strategies for all databases, registers and websites, including any filters and limits used.	Search strategy (page 2-3, line 85-99)
**Selection process**	8	Specify the methods used to decide whether a study met the inclusion criteria of the review, including how many reviewers screened each record and each report retrieved, whether they worked independently, and if applicable, details of automation tools used in the process.	Study selection (page 3, line 113-118)
**Data collection process**	9	Specify the methods used to collect data from reports, including how many reviewers collected data from each report, whether they worked independently, any processes for obtaining or confirming data from study investigators, and if applicable, details of automation tools used in the process.	Data extraction and quality assessment (page 3, line 119-142)

Topic	No.	Item	Location where item is reported
**Data items**	10a	List and define all outcomes for which data were sought. Specify whether all results that were compatible with each outcome domain in each study were sought (e.g. for all measures, time points, analyses), and if not, the methods used to decide which results to collect.	Data extraction and quality assessment (page 3, line 119-135)
	10b	List and define all other variables for which data were sought (e.g. participant and intervention characteristics, funding sources). Describe any assumptions made about any missing or unclear information.	Data extraction and quality assessment (page 3, line 119-135)
**Study risk of bias assessment**	11	Specify the methods used to assess risk of bias in the included studies, including details of the tool(s) used, how many reviewers assessed each study and whether they worked independently, and if applicable, details of automation tools used in the process.	Data extraction and quality assessment (page 3, line 136-142)
**Effect measures**	12	Specify for each outcome the effect measure(s) (e.g. risk ratio, mean difference) used in the synthesis or presentation of results.	Statistical Analysis (page 3, line 132-135)
**Synthesis methods**	13a	Describe the processes used to decide which studies were eligible for each synthesis (e.g. tabulating the study intervention characteristics and comparing against the planned groups for each synthesis (item 5)).	Statistical Analysis (page 4, line 143-162)
	13b	Describe any methods required to prepare the data for presentation or synthesis, such as handling of missing summary statistics, or data conversions.	Statistical Analysis (page 4, line 143-162)
	13c	Describe any methods used to tabulate or visually display results of individual studies and syntheses.	Statistical Analysis (page 4, line 159-162)
	13d	Describe any methods used to synthesize results and provide a rationale for the choice(s). If meta-analysis was performed, describe the model(s), method(s) to identify the presence and extent of statistical heterogeneity, and software package(s) used.	Statistical Analysis (page 4, line 143-162)
	13e	Describe any methods used to explore possible causes of heterogeneity among study results (e.g. subgroup analysis, meta-regression).	Statistical Analysis (page 4, line 143-162)
	13f	Describe any sensitivity analyses conducted to assess robustness of the synthesized results.	Statistical Analysis (page 4, line 158-159)

Topic	No.	Item	Location where item is reported
**Reporting bias assessment**	14	Describe any methods used to assess risk of bias due to missing results in a synthesis (arising from reporting biases).	Data extraction and quality assessment (page 4, line 161-162)
**Certainty assessment**	15	Describe any methods used to assess certainty (or confidence) in the body of evidence for an outcome.	Data Analysis and Statistical Analysis (page 4, line 136-162, Table S2 in Supplemental material)
**RESULTS**			
**Study selection**	16a	Describe the results of the search and selection process, from the number of records identified in the search to the number of studies included in the review, ideally using a flow diagram.	Study Characteristics (page 4-5, line 164-196), Figure 1 PRISMA flow
	16b	Cite studies that might appear to meet the inclusion criteria, but which were excluded, and explain why they were excluded.	Study Characteristics (page 4-5, line 164-196), Figure 1 PRISMA flow
**Study characteristics**	17	Cite each included study and present its characteristics.	Table 1
**Risk of bias in studies**	18	Present assessments of risk of bias for each included study.	Methodological quality (page 5, line 197-204), Figure S1 in Supplemental material
**Results of individual studies**	19	For all outcomes, present, for each study: (a) summary statistics for each group (where appropriate) and (b) an effect estimate and its precision (e.g. confidence/credible interval), ideally using structured tables or plots.	Results (page 8-12, line 214-300), Table 2, Figure 2-5
**Results of syntheses**	20a	For each synthesis, briefly summarise the characteristics and risk of bias among contributing studies.	Results (page 8-12, line 214-299), Table 2, Figure 2-5
	20b	Present results of all statistical syntheses conducted. If meta-analysis was done, present for each the summary estimate and its precision (e.g. confidence/credible interval) and measures of statistical heterogeneity. If comparing groups, describe the direction of the effect.	Results (page 8-12, line 214-299), Table 2, Figure 2-5
	20c	Present results of all investigations of possible causes of heterogeneity among study results.	Results (page 8-12, line 214-299), Table 2, Figure 2-5
	20d	Present results of all sensitivity analyses conducted to assess the robustness of the synthesized results.	Sensitivity analysis (page 12, line 300-304), Table S3 in Supplemental material

Topic	No.	Item	Location where item is reported
**Reporting biases**	21	Present assessments of risk of bias due to missing results (arising from reporting biases) for each synthesis assessed.	Results (page 12, line 305-313), Figure 6
**Certainty of evidence**	22	Present assessments of certainty (or confidence) in the body of evidence for each outcome assessed.	Results (page 8-12, line 214-299), Table S2 in Supplemental material, Figure 2-5
**DISCUSSION**			
**Discussion**	23a	Provide a general interpretation of the results in the context of other evidence.	Discussion (page 13-16, line 316-482)
	23b	Discuss any limitations of the evidence included in the review.	Discussion (page 13-16, line 464-482)
	23c	Discuss any limitations of the review processes used.	Discussion (page 13-16, line 464-482)
	23d	Discuss implications of the results for practice, policy, and future research.	Discussion and Conclusions (page 15-16, line 447-463 and 479-494)
**OTHER INFORMATION**			
**Registration and protocol**	24a	Provide registration information for the review, including register name and registration number, or state that the review was not registered.	Search strategy (page 2, line 86-87)
	24b	Indicate where the review protocol can be accessed, or state that a protocol was not prepared.	Search strategy (page 2-3, line 86-87)
	24c	Describe and explain any amendments to information provided at registration or in the protocol.	Search strategy (page 3, line 86-99)
**Support**	25	Describe sources of financial or non-financial support for the review, and the role of the funders or sponsors in the review.	Funding (page 16, line 506)
**Competing interests**	26	Declare any competing interests of review authors.	Conflict of interest (page 16, line 512)
**Availability of data, code and other materials**	27	Report which of the following are publicly available and where they can be found: template data collection forms; data extracted from included studies; data used for all analyses; analytic code; any other materials used in the review.	Data Availability Statement (page 16, line 510-511)

Table S2. Search terms

1	Kidney transplant*.mp. [mp=ti, ab, hw, tn, ot, dm, mf, dv, kf, fx, dq, bt, nm, ox, px, rx, ui, sy, ux, mx, sh, kw, tx, ct]
2	Renal transplant*.mp. [mp=ti, ab, hw, tn, ot, dm, mf, dv, kf, fx, dq, bt, nm, ox, px, rx, ui, sy, ux, mx, sh, kw, tx, ct]
3	1 or 2
4	Death.mp. [mp=ti, ab, hw, tn, ot, dm, mf, dv, kf, fx, dq, bt, nm, ox, px, rx, ui, sy, ux, mx, sh, kw, tx, ct]
5	Mortality.mp. [mp=ti, ab, hw, tn, ot, dm, mf, dv, kf, fx, dq, bt, nm, ox, px, rx, ui, sy, ux, mx, sh, kw, tx, ct]
6	Acute kidney injury.mp. [mp=ti, ab, hw, tn, ot, dm, mf, dv, kf, fx, dq, bt, nm, ox, px, rx, ui, sy, ux, mx, sh, kw, tx, ct]
7	Acute renal injury.mp. [mp=ti, ab, hw, tn, ot, dm, mf, dv, kf, fx, dq, bt, nm, ox, px, rx, ui, sy, ux, mx, sh, kw, tx, ct]
8	Acute kidney failure.mp. [mp=ti, ab, hw, tn, ot, dm, mf, dv, kf, fx, dq, bt, nm, ox, px, rx, ui, sy, ux, mx, sh, kw, tx, ct]
9	Acute renal failure.mp. [mp=ti, ab, hw, tn, ot, dm, mf, dv, kf, fx, dq, bt, nm, ox, px, rx, ui, sy, ux, mx, sh, kw, tx, ct]
10	Acute kidney insufficiency.mp. [mp=ti, ab, hw, tn, ot, dm, mf, dv, kf, fx, dq, bt, nm, ox, px, rx, ui, sy, ux, mx, sh, kw, tx, ct]
11	Acute renal insufficiency.mp. [mp=ti, ab, hw, tn, ot, dm, mf, dv, kf, fx, dq, bt, nm, ox, px, rx, ui, sy, ux, mx, sh, kw, tx, ct]
12	AKI.mp. [mp=ti, ab, hw, tn, ot, dm, mf, dv, kf, fx, dq, bt, nm, ox, px, rx, ui, sy, ux, mx, sh, kw, tx, ct]
13	Graft failure.mp. [mp=ti, ab, hw, tn, ot, dm, mf, dv, kf, fx, dq, bt, nm, ox, px, rx, ui, sy, ux, mx, sh, kw, tx, ct]
14	Graft rejection.mp. [mp=ti, ab, hw, tn, ot, dm, mf, dv, kf, fx, dq, bt, nm, ox, px, rx, ui, sy, ux, mx, sh, kw, tx, ct]
15	4 or 5 or 6 or 7 or 8 or 9 or 10 or 11 or 12 or 13 or 14
16	Intensive care.mp. [mp=ti, ab, hw, tn, ot, dm, mf, dv, kf, fx, dq, bt, nm, ox, px, rx, ui, sy, ux, mx, sh, kw, tx, ct]
17	Critical care.mp. [mp=ti, ab, hw, tn, ot, dm, mf, dv, kf, fx, dq, bt, nm, ox, px, rx, ui, sy, ux, mx, sh, kw, tx, ct]
18	Critical illness.mp. [mp=ti, ab, hw, tn, ot, dm, mf, dv, kf, fx, dq, bt, nm, ox, px, rx, ui, sy, ux, mx, sh, kw, tx, ct]
19	Critically ill.mp. [mp=ti, ab, hw, tn, ot, dm, mf, dv, kf, fx, dq, bt, nm, ox, px, rx, ui, sy, ux, mx, sh, kw, tx, ct]
20	ICU.mp. [mp=ti, ab, hw, tn, ot, dm, mf, dv, kf, fx, dq, bt, nm, ox, px, rx, ui, sy, ux, mx, sh, kw, tx, ct]
21	CCU.mp. [mp=ti, ab, hw, tn, ot, dm, mf, dv, kf, fx, dq, bt, nm, ox, px, rx, ui, sy, ux, mx, sh, kw, tx, ct]
22	16 or 17 or 18 or 19 or 20 or 21
23	3 and 15 and 22
24	limit 23 to humans
25	remove duplicates from 24

<u>Table S3.</u> Summary of findings and certainty assessment in accordance to GRADE framework

Certainty assessment							Summary of findings				
Participants (studies) Follow-up	Risk of bias	Inconsistency	Indirectness	Imprecision	Publication bias	Overall certainty of evidence	Study event rates (%)		Relative effect (95% CI)	Anticipated absolute effects	
							With non-transplant recipient	With Kidney transplant recipient		Risk with non-transplant recipient	Risk difference with Kidney transplant recipient
**Overall mortality (assessed with: Odds ratio)**											
12062 (7 non-randomised studies)	serious[a]	serious[b]	not serious	serious[c]	all plausible residual confounding would reduce the demonstrated effect	⊕⊕◯◯ Low[a,b,c]	2922/11602 (25.2%)	126/460 (27.4%)	**OR 1.82** (0.79 to 4.16)	2922/11602 (25.2%)	**128 more per 1,000** (from 42 fewer to 332 more)
**ICU mortality (assessed with: Odds ratio)**											
11669 (5 non-randomised studies)	serious[a]	serious[b]	not serious	serious[d]	all plausible residual confounding would reduce the demonstrated effect	⊕⊕◯◯ Low[a,b,d]	2878/11314 (25.4%)	76/355 (21.4%)	**OR 1.06** (0.45 to 2.48)	2878/11314 (25.4%)	**11 more per 1,000** (from 121 fewer to 204 more)
**28/30-day mortality (assessed with: Odds ratio)**											
502 (2 non-randomised studies)	serious[a]	serious[b]	not serious	serious[c,d]	all plausible residual confounding would reduce the demonstrated effect	⊕⊕◯◯ Low[a,b,c,d]	88/365 (24.1%)	56/137 (40.9%)	**OR 2.06** (0.30 to 14.10)	88/365 (24.1%)	**154 more per 1,000** (from 154 fewer to 576 more)
**1-year mortality (assessed with: Odds ratio)**											
114 (1 non-randomised study)	not serious	not serious	not serious	not serious	very strong association	⊕⊕⊕⊕ High	3/76 (3.9%)	8/38 (21.1%)	**OR 6.49** (1.61 to 26.23)	3/76 (3.9%)	**171 more per 1,000** (from 23 more to 479 more)
**5-year mortality (assessed with: Odds ratio)**											
114 (1 non-randomised study)	not serious	not serious	not serious	not serious	strong association	⊕⊕⊕⊕ High	7/76 (9.2%)	14/38 (36.8%)	**OR 5.75** (2.08 to 15.87)	7/76 (9.2%)	**276 more per 1,000** (from 82 more to 525 more)
**ICU length of stay (assessed with: Weighted mean difference)**											
7659 (6 non-randomised studies)	serious[a]	not serious	not serious	not serious	all plausible residual confounding would reduce the demonstrated effect	⊕⊕⊕⊕ High[a]	7295	364	-	7295	MD **1.96 days more** (0.81 more to 3.11 more)
**Need for renal replacement therapy (assessed with: Odds ratio)**											
616 (3 non-randomised studies)	serious[a]	serious[b]	not serious	serious[d]	all plausible residual confounding would reduce the demonstrated effect	⊕⊕◯◯ Low[a,b,d]	141/441 (32.0%)	70/175 (40.0%)	**OR 2.20** (0.53 to 9.07)	141/441 (32.0%)	**189 more per 1,000** (from 120 fewer to 490 more)
**Need for inotropic drugs (assessed with: Odds ratio)**											
616 (3 non-randomised studies)	serious[a]	not serious	not serious	serious[c,d]	all plausible residual confounding would reduce the demonstrated effect	⊕⊕⊕◯ Moderate[a,c,d]	216/441 (49.0%)	70/175 (40.0%)	**OR 0.78** (0.52 to 1.16)	216/441 (49.0%)	**62 fewer per 1,000** (from 157 fewer to 37 more)
**Need for mechanical ventilation (assessed with: Odds ratio)**											
502 (2 non-randomised studies)	serious[a]	serious[b]	not serious	serious[c,d]	all plausible residual confounding would reduce the demonstrated effect	⊕⊕◯◯ Low[a,b,c,d]	227/365 (62.2%)	71/137 (51.8%)	**OR 0.73** (0.33 to 1.62)	227/365 (62.2%)	**76 fewer per 1,000** (from 270 fewer to 105 more)
**New onset infection (assessed with: Odds ratio)**											
279 (1 non-randomised study)	not serious	not serious	not serious	not serious	all plausible residual confounding would reduce the demonstrated effect	⊕⊕⊕⊕ High	70/212 (33.0%)	16/67 (23.9%)	**OR 0.64** (0.34 to 1.20)	70/212 (33.0%)	**90 fewer per 1,000** (from 187 fewer to 41 more)
**SOFA score (assessed with: Weighted mean difference)[e]**											
337 (2 non-randomised studies)	serious[a]	not serious	not serious	serious[c,d]	all plausible residual confounding would reduce the demonstrated effect	⊕⊕⊕◯ Moderate[a,c,d]	210	127	-	210	MD **0.79 more** (0.78 fewer to 2.36 more)

CI: confidence interval; **MD:** mean difference; **OR:** odds ratio

Explanations

a. Certain studies contain moderate risk of confounding.
b. High heterogeneity
c. Small total number of events
d. Wide confidence intervals (CI) include both no effect and appreciable benefit and harm.
e. SOFA, the Sequential Organ Failure Assessment score

Table S4. Sensitivity analysis (leave-one-out method) represented by funnel plot of pooled odds ratio from included studies omitting one study identified by the name of first author; CI, confidence interval

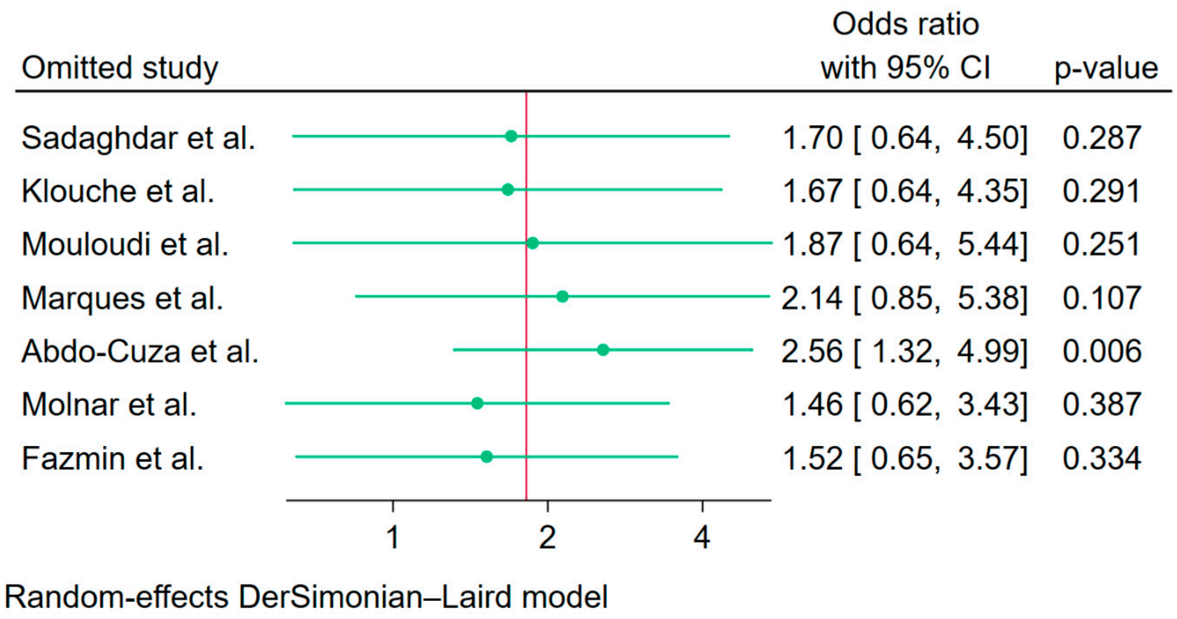

Figure S1. Summary of Risk of Bias in Non-randomized Studies – of Interventions (ROBIN-I) V2 for included studies

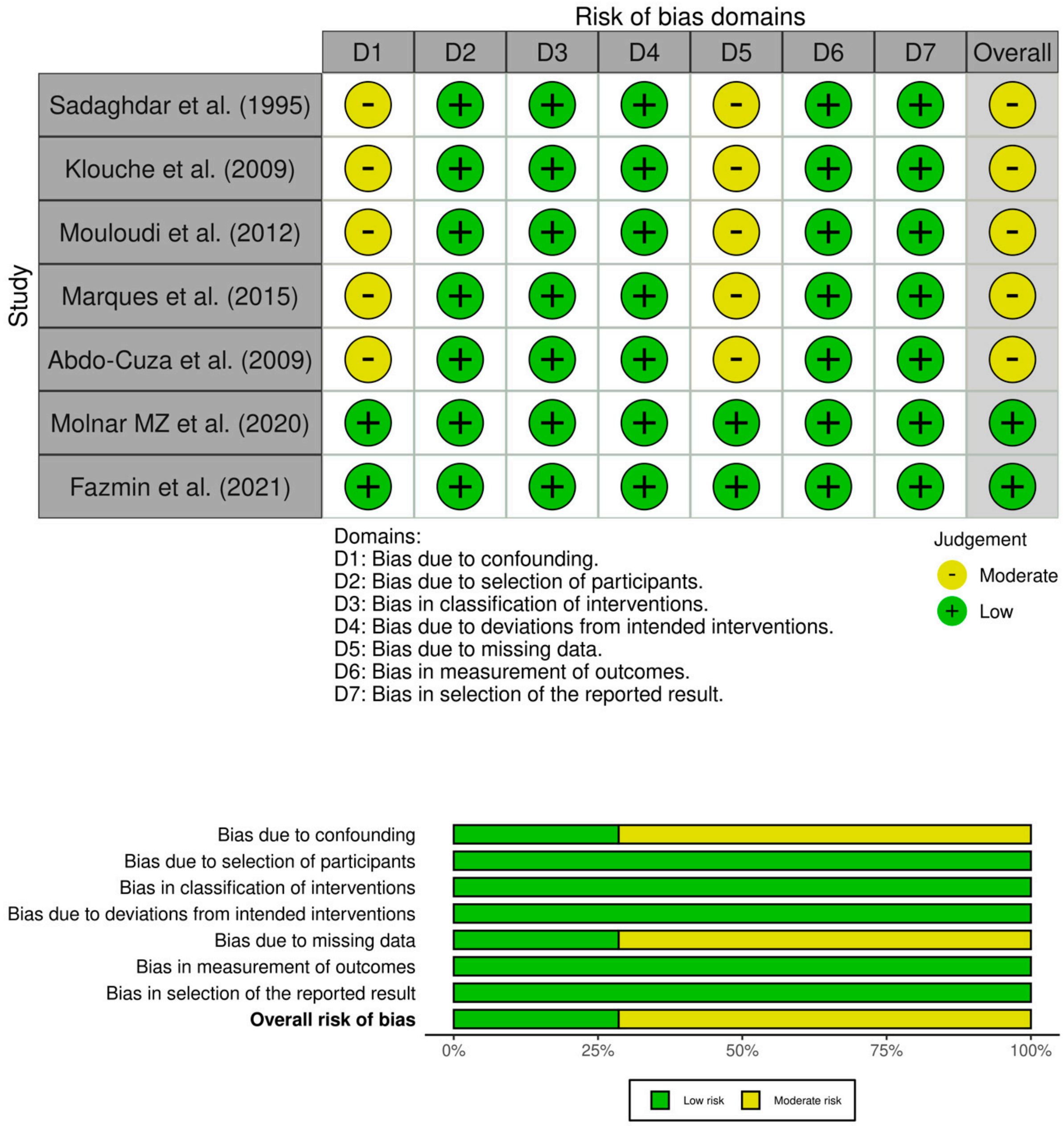

Supplement: Supplementary file 1 [file jcm-14-02284-s001.zip › jcm-3482797-supplementary.pdf]
